# Ozone

**Published:** 1878-02

**Authors:** 


OZONE:

Let us also for a moment consider the ozone in the air, which may be looked upon as polarized or agitated oxygen. After its discovery, which has immortalized the name of Schonbein, was made known, it was thought for a time that the key had been found for the appearance and disappearance of various diseases, in the quantity of ozone in the air. But one fact, which was observed from the first, shows that it cannot be so; for the presence of ozone can never be detected in our dwellings, not even in the cleanest and best ventilated. Now, as it is a fact that we spend the greater part of our lives in our houses, and are better than if we lived in open air, the hygienic value of ozone does not seem so very great. Added to this, the medical men of Konigsberg long had several ozone-stations there, during which time various diseases came and went, without, as appears from the reports of Dr. Schiefferdecker, ozone having the slightest connection with the appearance or disappear- of any of them.

Dr. Wolfhugel, assistant at the Hygienic Institute at Munich, has lately been occupied with the question of the sanitary value of ozone, but has arrived at only negative results.—*Scribner's Monthly*, for February.

